# Editorial: Symbiotic organs in insects: development, metabolism, and physiological regulation

**DOI:** 10.3389/fphys.2023.1248654

**Published:** 2023-07-05

**Authors:** Federica Calevro, Patrick Callaerts, Yu Matsuura, Anna Michalik

**Affiliations:** ^1^ INRAE, INSA Lyon, BF2I, UMR 203, Université de Lyon, Villeurbanne, France; ^2^ Department of Human Genetics, Katholieke Universiteit Leuven, Leuven, Belgium; ^3^ Tropical Biosphere Research Center, University of the Ryukyus, Nishihara, Okinawa, Japan; ^4^ Department of Developmental Biology Morphology of Invertebrates, Faculty of Biology, Institute of Zoology and Biomedical Research, Jagiellonian University, Krakow, Poland

**Keywords:** insects, bacterial symbionts, bacteriocytes, gut symbiosis, insect glands, development, anatomy, symbiont transmission

Associations with microbial symbionts enable insects to thrive on nutritionally unbalanced diets or in adverse environmental conditions (Baumann, 2005; Douglas, 2015). These beneficial symbionts can live intracellularly, inside specialized host cells, or extracellularly, in expansions or modifications of the host organs. These structures are biological innovations, pivotal for maintaining symbionts through host generations. Studies are needed to elucidate the mechanisms underlying their development and morphogenesis, their anatomical diversity, and their integration in host physiology. This research topic was designed to cover those aspects. It contains seven original articles and one review, expected to inform, and inspire researchers in the field.

Four papers focus on bacteriocytes/bacteriomes. These cells/organs are specialized in harboring microbial symbionts (Buchner, 1965), and have emerged independently in several insect orders. Their localization differs depending on insect physiology and the evolutionary history of the association. In the weevil *Sitophilus oryzae* (Coleoptera: Curculionidea), with the recently acquired endosymbiont *Sodalis pierantonius*, somatic bacteriomes are eliminated in adults by apoptosis and autophagy (Vigneron et al., 2014), once the symbiont has provided tyrosine for cuticle formation. Conversely, ovarian bacteriomes are present in all stages, as they are involved in maternal symbiont transmission. The work of Ferrarini et al. shows how endosymbionts colonize the germarium in one-week-old *Sitophilus* females. Transcriptomic analysis of weevil ovarian bacteriomes shows that immune effectors are downregulated at the onset of sexual maturation, suggesting that relaxation of endosymbiont control by antimicrobial peptides allows bacterial migration and oocyte infection. Aphids (Hemiptera: Aphididae) have evolved a long-lasting association with the obligatory, metabolically highly integrated gammaproteobacterium *Buchnera aphidicola.* Symbionts are present throughout the aphid life cycle, and bacteriocyte and endosymbiont dynamics are highly coordinated and regulated by processes involving novel cell death mechanisms (Simonet et al., 2018). Ribeiro Lopes et al. show that aphid bacteriocyte numbers and sizes, and cell death processes they undergo, are remarkably plastic and vary in an environmental stress-specific manner (amino acid stress, starvation). This suggests that bacteriocytes are more than a privileged environment to house symbionts, and that studies are needed to understand the crosstalk between these cells and the rest of the body. Michalik et al. explored the diversity of heritable symbionts in planthoppers (Hemiptera: Fulgoromorpha). This hemipteran group presents very complex symbiotic systems. It is associated with ancestral symbionts *Sulcia* and *Vidania* (Michalik et al., 2021) which, in many lineages, are accompanied or have been replaced by other heritable bacteria (e.g., *Sodalis*, *Arsenophonus*, *Purcelliella*) or yeast-like fungi. The authors compared symbiont tissue distributions and bacteriome organization in 15 planthopper families. They found at least seven different types of bacteriocytes, with symbionts also colonizing the cytoplasm or the nuclei of the fat body, the rectal organ, or the hemolymph and the gut lumen. This correlates with the acquisition, replacement patterns, and functions of symbionts. The review by Alarcón et al. recalls the history of bacteriocyte discovery across insect orders, summarizes the profound molecular changes potentially brought about by acquisitions of symbionts and the diversity of these novel cells, and discusses their evolutionary developmental origin and ontogeny.

More recently emerged symbionts tend to be less dependent on host tissues and can be housed extracellularly. In phytophagous stinkbugs (Hemiptera: Pentatomomorpha), the symbionts are housed in sac-like structures in the posterior midgut. Stinkbugs can bear vertically transmitted Gammaproteobacteria (Fukatsu and Hosokawa, 2002) or Betaproteobacteria horizontally acquired at each new generation (Kikuchi et al., 2011). The study of Moriyama and Fukatsu focuses on the plataspid stinkbug *Megacopta punctatissima,* and its obligatory Gammaproteobacteria “Candidatus *Ishikawella capsulata*”. *Ishikawella* has lost some metabolic pathways redundant with the host and retained pathways involved in the production of essential amino acids, suggesting a potential role as a nutritional symbiont. The authors characterized the effect of symbiont removal on essential amino acid content and measured amino acid production in symbiotic organs cultured *in vitro*, then demonstrating, for the first time, that an extracellular symbiont functions similarly to nutritional symbionts residing in bacteriocytes. They establish a framework to study the mechanistic basis of these associations. Jang et al. focus on the association between the bean bug *Riptortus terrestris* and the betaproteobacteria *Caballeronia*. These authors demonstrate that symbiont colonization activates stem cell proliferation in the symbiont-bearing crypts and prolongs cellular life span of their epithelial cells by suppressing apoptosis, thereby establishing the drastic morphological changes that result in a stable symbiotic association. This opens the way for studies aimed at identifying the molecular mechanisms and signals regulating crypt morphogenesis in stinkbug symbioses.

Janke et al. focus on the association between *Lagria* darkling beetles (Coleoptera: Tenebrionoidea) and the community of symbionts (dominated by *Burkholderia*) colonizing the female accessory glands (Floréz and Kaltenpoth, 2017). The symbionts produce antibiotics that protect eggs and larvae against fungal infection. The authors describe how symbiont transmission occurs externally, via the host surface, from female pupae to adults. They also show that symbiont loss in males begins at the pupal stage, and is accompanied by morphological changes of the symbiotic organ during metamorphosis. The association with symbiotic bacteria capable of protecting offspring from fungal infection is not unique to beetles. This also constitutes the basis of the association between solitary digger wasps of the tribe *Philanthini*, (Hymenoptera: Crabronidae) and *Streptomyces* bacteria. Here the symbionts are housed in antennal glands (Kaltenpoth et al., 2014). Goettler et al. investigated the morphology of antennal glands of 14 *Philanthus* species from the Palearctic, Paleotropic, and Nearctic. They combined 3D-models of the glands with phylogenetic analyses and found that, despite their overall similarity, the morphology of the glands differs gradually across species, largely related to geographic origin and phylogenetic position (African and European species showing more complex structures compared to North-American species). These results corroborate the hypothesis that new-world and old-world species represent distinct subgroups within this genus.

We believe that the availability of high-quality insect genome sequences and of new imaging technologies constitutes a unique opportunity for in-depth and comparative studies into i) the evolutionary developmental origin of symbiotic organs across species, ii) the ways in which symbionts subvert cellular mechanisms to invade and colonize host tissues, and iii) how symbiotic organs are integrated in and contribute to host physiology and metabolism.
